# Identification of Serogroups Australis and Icterohaemorrhagiae in Two Dogs with a Severe Form of Acute Leptospirosis in Italy

**DOI:** 10.3390/pathogens9050351

**Published:** 2020-05-06

**Authors:** Andrea Balboni, Silvia Zamagni, Cristina Bertasio, Maria Beatrice Boniotti, Roberta Troìa, Mara Battilani, Francesco Dondi

**Affiliations:** 1 Department of Veterinary Medical Sciences, Alma Mater Studiorum, University of Bologna, 40064 Ozzano dell’Emilia, Bologna, Italy; a.balboni@unibo.it (A.B.); silvia.zamagni5@unibo.it (S.Z.); roberta.troia2@unibo.it (R.T.); f.dondi@unibo.it (F.D.); 2 National Reference Centre for Animal Leptospirosis (NRCL), Istituto Zooprofilattico Sperimentale della Lombardia ed Emilia Romagna “Bruno Ubertini”, via Bianchi 7/9, 25121 Brescia, Italy; cristina.bertasio@izsler.it (C.B.); mariabeatrice.boniotti@izsler.it (M.B.B.)

**Keywords:** Australis, canine leptospirosis, Icterohaemorrhagiae, multi-locus sequence typing

## Abstract

Leptospirosis is an infectious disease that causes serious illness in dogs. For this reason, epidemiological and clinical studies focusing on disease characterization are widely advocated. The aim of this study was to characterize the leptospires identified in dogs with confirmed symptomatic acute leptospirosis. *Leptospira* spp. DNA detected in urine, blood, or both samples from nine infected dogs was analyzed using the multi-locus sequence typing (MLST) technique. Leptospires from two dogs were successfully typed: one was identified as belonging to Sequence Type (ST) 17 and one to ST198, both within the *L. interrogans* species, serogroups Icterohaemorrhagiae and Australis, respectively. Based on the results of routine serologic tests, antibodies reactive toward these serogroups are commonly revealed in dogs in Italy. This study provides the first molecular analysis that identifies infecting *Leptospira* directly on DNA from biological samples of dogs, showing that serogroup Australis can lead to a severe clinical presentation of leptospirosis in infected dogs.

## 1. Introduction

Leptospirosis is a worldwide zoonotic disease affecting many mammalian species [1]. Leptospiral infection in dogs causes severe clinical manifestations, which can lead to death in 28–70% of cases [2,3]. In Europe, the serological prevalence of leptospirosis in dogs is about 25% [4,5]. A similar prevalence (29.9%) is reported in Italy according to a study performed in the whole Italian area [6], while the prevalence reported in a more recent paper in North-Central Italy was 8.65% [7]. Despite the high serological prevalence of the infection, data obtained by molecular typing techniques to identify *Leptospira* strains are lacking in our country.

The diagnosis of canine leptospirosis is frequently carried out with serological tests such as the microagglutination test (MAT) performed upon admission or in paired serum samples, as previously recommended [8]. According to the available literature, MAT on serum samples is not able to appropriately recognize the infecting serovar, being able to only identify the serogroup. The serogroup with the highest MAT titer is generally considered the infecting one [9]. This MAT interpretation, however, can lead to flawed conclusions: due to the presence of common antigens among serogroups and the potential for in vitro cross-reaction, several serogroups with high antibody titers can sometimes be displayed by the same dog [10]. Moreover, the serogroup with the highest MAT titer frequently changes over time and between different laboratories, suggesting that it might not really represent the infecting serogroup [10].

PCR or real-time PCR (qPCR) is often carried out to detect *Leptospira* spp. DNA in a variety of biological samples and to diagnose leptospirosis in dogs. Although molecular tests showed low diagnostic sensitivity as they might give a large number of false negative results [11], different techniques have been adopted for the typing of *Leptospira* strains by analyzing the bacterial genome or its specific regions [12,13,14,15]. In particular, the multi-locus sequence typing (MLST) technique is able to characterize the genetic profile of *Leptospira* strains by sequencing and analyzing specific fragments of some bacterial house-keeping genes, thus identifying specific sequence types (STs).

The aim of this study was to characterize by MLST analysis the DNA of leptospires detected in dogs affected by acute leptospirosis.

## 2. Results

**Figure 1:**
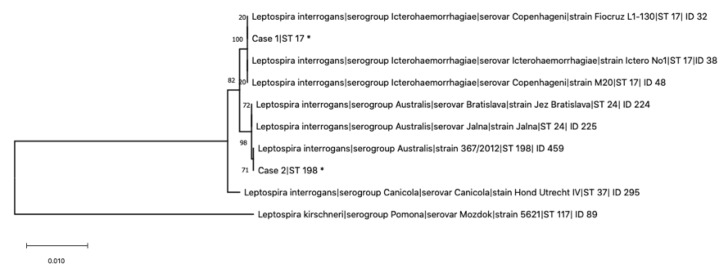
Maximum likelihood tree built on concatenated sequences of the seven multi-locus sequence typing (MLST) loci (3111 bp) of the scheme proposed by Boonsilp and colleagues [16]. Phylogeny was conducted in MEGA X using the Tamura–Nei model, and bootstrap values are indicated on the respective branches. Additional sequences included in the alignment were retrieved from the Pubmlst database. * indicates the leptospires genotyped in this study from dogs of Case 1 (Sequence Type 17 (ST17)) and Case 2 (ST198).

Blood and urine samples from nine dogs with acute leptospirosis and with a positive qPCR were used for the study. These dogs were part of a previous study on leptospirosis conducted by our research group [11]. Six out of nine dogs were intact males, 2/9 were spayed females, and 1/9 was an intact female. The median age was four years (range 1–12). Three out of nine dogs were mixed-breed; 2/9 were Labrador retriever; and the remaining 4/9 were Jack Russell terrier, German Shepherd, Weimaraner, Leonberger, and Kurzhaar. Seven out nine dogs had an outdoor lifestyle, whereas only 2/9 had an urban lifestyle. Five out of nine dogs had been vaccinated with a bivalent vaccine, while 4/9 had not been correctly vaccinated. Finally, 4/9 dogs survived, while 5/9 died or were humanely euthanized. The MLST analysis was performed using the scheme proposed by Boonsilp and colleagues [16] on the DNA extracted from the included samples. A complete MLST profile was obtained from a blood sample and from a urine sample belonging to two dogs, Case 1 and Case 2, respectively (GenBank ID: MT411548-MT411561). In Case 1, the infecting Leptospira belonged to ST17, while in Case 2, the genotype of the infecting Leptospira was ST198 (Figure 1). We were unable to achieve a successful PCR amplification in MLST loci in the samples from the remaining seven dogs, probably due to the low amount of leptospiral DNA present.

*Leptospira* from Case 1 clustered with international strains characterized as *L. interrogans* serogroup Icterohaemorrhagiae, serovars Icterohaemorrhagiae and Copenhageni, while *Leptospira* from Case 2 clustered with strain 367/2012, reported as *L. interrogans* serogroup Australis (Figure 1). As depicted in Figure 1, ST198 resulted in being very similar to ST24, found in *L. interrogans* Jalna and Bratislava, having all identical alleles except for the pntA gene, which showed the substitution 234C-T and was registered in the Pubmlst database as “allele 66”.

Both Case 1 and Case 2 had a severe and acute clinical presentation of the disease with acute kidney injury (AKI), systemic inflammation, and evidence of damage/dysfunction of at least one organ other than the kidneys. Case 1 survived and was discharged from the hospital after ten days of hospitalization. This was a one-year old, intact male Weimaraner. MAT was negative at the time of admission, but a positive convalescent MAT was evaluated after seven days from presentation (1:400 serogroup Icterohaemorrhagiae, 1:200 serogroup Canicola). Initial diagnosis was carried out with qPCR from blood collected upon admission, whereas the concurrent urine sample was negative. Case 2 died 24 h after hospitalization. This dog was a twelve-year old, spayed female Labrador retriever. The patient was negative for MAT upon admission, and no convalescent test was performed due to the early death of the patient. The qPCR was positive both for blood and urine samples. Neither of these two cases had been properly vaccinated, as previously recommended [17], and both had an outdoor lifestyle, with free access to swampy areas and, potentially, to wild reservoirs of leptospirosis.

## 3. Discussion

In this study, we performed the molecular characterization of the *Leptospira* infecting two dogs with a confirmed diagnosis of acute leptospirosis. In Case 1, the infecting *Leptospira* belonged to ST17, related to *L. interrogans* serogroup Icterohaemorrhagiae, while Case 2 resulted in being affected by *Leptospira* ST198 related to *L. interrogans* serogroup Australis. Based on the MAT results, it is quite common to reveal antibodies reactive toward these serogroups in dogs in Italy [6,7]. Attempts to deduce the infecting serogroup from serological data were made, but they were subjected to several limitations, as previously stated. Hence, tests able to type *Leptospira* strains correctly would allow better detailing the epidemiology of the disease in different geographic areas, to target the need for specific vaccines, as well as to highlight potential serogroup-specific clinical pictures and prognostic factors in affected animals. This is the first time that the MLST technique has been applied directly to biological samples from dogs in Italy, allowing obtaining *Leptospira* typing before the onset of an IgG response detectable by MAT. In a recent study performed in Brazil, MLST was carried out on two *Leptospira* strains isolated from canine urine samples identifying *L. interrogans* serogroup Icterohaemorrhagiae [12]. A larger study performed in Japan applied the MLST technique on 45 strains isolated from dogs with acute leptospirosis, identifying 16/45 strains belonging to serogroup Australis and 1/45 to serogroup Icterohaemorrhagiae [13]. *L. interrogans* serogroup Australis was lethal in most of those cases (78.6%). Afterwards, the same Japanese working group confirmed these previous data on dogs, identifying 21/75 leptospiral isolates belonging to serogroup Australis, with a mortality rate related to this serogroup as high as 83.3% [14].

In Europe, data regarding the application of MLST in dogs with leptospirosis are lacking. In a recent study, MLST was applied to characterize *L. interrogans* isolated from wild, domestic, and captive animals in Portugal [18]. Serovar Copenhageni (serogroup Icterohaemorrhagiae) was detected in five distinct captive animals and in one rat from Lisbon zoo, and serovar Bratislava (serogroup Australis) was detected in one healthy horse. Another study performed in Northern Ireland demonstrated the presence of *L. interrogans* serogroup Australis in 15/35 (42.7%) horses using MLST [19]. Furthermore, the authors of the cited study compared these results with the frequency of the serogroup Australis in other herbivores (sheep 6.9% and cattle 0.9%).

As is common knowledge, *L. interrogans* serogroup Icterohaemorrhagiae is associated with severe clinical signs, whereas data regarding the pathogenicity of the Australis serogroup and its clinical features during leptospirosis in dogs are limited [3,11,13,14,20,21]. Interestingly, ST198 found in Case 2 was firstly discovered in hedgehogs of Northern Italy by Boniotti and colleagues [22]. Its detection in this study suggests a possible direct transmission between different species or an indirect infection through a contaminated shared environment. Moreover, the current study revealed that ST198 was diffused in Italy and associated with a severe life-threating clinical presentation with multiorgan involvement. A severe clinical presentation in dogs associated with infection with a *Leptospira* ST related to *L. interrogans* serogroup Australis was previously reported in Asia [13,14].

Different types of vaccines are available in Italy: bivalent vaccines containing two serovars (Canicola and Icterohaemorrhagiae), a trivalent vaccine containing serovars Canicola, Icterohaemorrhagiae, and Grippotyphosa, and tetravalent vaccines containing Canicola, Icterohaemorrhagiae, Grippotyphosa, and Bratislava, which belong to the Australis serogroup. Despite the vaccination against serogroup Icterohaemorrhagiae having been used for many years in Italy, this study proves that this serogroup is still circulating and able to infect non-vaccinated dogs. Thus, according to the results reported in this paper, vaccination against both of these serogroups (Icterohaemorrhagiae and Australis) is recommended in Italy.

MLST is currently adopted to correlate leptospiral STs with serotyping; nevertheless, according to the available literature, immunity stimulated by vaccination seems to be serovar-specific [9]. Furthermore, Koizumi end collaborators (2013) suggested that in some cases, MLST is not suitable for identifying serogroups, since it may detect the same ST in more than one serogroup [13]. The same authors compared MLST with multi-locus variable-number tandem repeat analysis (MLVA), concluding that the latter one had a higher discriminatory power and was more concordant with serotyping [14]. Therefore, further studies are needed to develop MLST protocols able to correlate unambiguously STs and serotyping and to identify the serovars belonging to serogroups Australis (serovars Bratislava, Australis, Lora, Muenchen) [23] spread throughout the Italian and European area, in order to target the research on leptospiral vaccines based on circulating serovars.

## 4. Materials and Methods

### 4.1. Sample Collection, Inclusion Criteria, and Diagnosis of Leptospirosis

Dogs included in the present study originated from a previous prospective study on canine acute leptospirosis carried out by Troìa and colleagues [11]. In that study, dogs with suspected acute leptospirosis were enrolled on the basis of reported exposure to known risk factors, clinical and clinicopathological signs suggesting AKI, systemic inflammation, or both, and evidence of dysfunction of at least one organ other than the kidney. The diagnosis of leptospirosis was confirmed by a combination of tests: MAT titer at the time of hospital admission (considered positive if the titer was ≥1:800), convalescent MAT titer (fourfold increase from baseline), and/or a positive qPCR from blood, urine samples, or both. Dogs vaccinated for leptospirosis within 15 weeks before hospitalization were excluded from the study [11]. DNA extracted from blood and/or urine samples of nine dogs testing positive for the presence of *Leptospira* spp. DNA using an SYBR Green qPCR was used for the following molecular analyses [11,24]. Four out of 9 dogs had positive blood samples; 4/9 dogs had positive urine samples; and 1/9 dogs had positive blood and urine samples with a *Leptospira* spp. DNA quantity ranging from 1.00 × 10^0^ to 2.09 × 10^2^ DNA copies/µL of extract (median 2.22 × 10^0^) [11].

### 4.2. Multi-Locus Sequence Typing

The analysis was performed using the seven loci (glmU, pntA, sucA, tpiA, pfkB, mreA, and caiB) scheme proposed by Boonsilp and colleagues [16] following the protocol previously developed for its application directly on DNA extracted from biological samples [25]. Nucleotide sequences for each of the seven genes were trimmed to the reference sizes using the module SeqMan of the Lasergene sequencing analysis software package (DNASTAR, Inc., Madison, WI, USA), and STs were assigned through the Pubmlst database [26]. Nucleotide sequences for each of the seven genes were concatenated and aligned using MegAlign software (Lasergene, DNASTAR). Additional sequences included in the alignment were retrieved from the Pubmlst database, and a phylogenetic tree was conducted in MEGA X Version 10.1.7 using the maximum likelihood method and the Tamura–Nei model with a bootstrap analysis based on 1000 replicates [27,28].

## 5. Conclusions

This study used the MLST technique and identified two different *L. interrogans* STs, corresponding to serogroups Icterohaemorrhagiae and Australis, respectively, in two dogs with clinical acute leptospirosis. This was the first time that MLST was applied in Italy to characterize *Leptospira* spp. directly on DNA from biological samples of symptomatic dogs, allowing typing *Leptospira* before the onset of an IgG response detectable by MAT, and independently of the availability of the isolated sample, which requires several days. The MLST technique appeared to be a valuable tool to overcome the limitations of serological testing for epidemiologic analysis of leptospirosis in different countries. In this regard, the results of the current study confirmed that the Australis serogroup can cause a form of acute leptospirosis with a severe clinical picture and supported the development of vaccination protocols with serovars belonging to this serogroup.
